# A Real-World, Single-Center, Observational Retrospective Experience of Durvalumab Treatment After Concomitant Chemoradiation for Unresectable Stage III Non-Small Cell Lung Cancer

**DOI:** 10.3390/cancers18061044

**Published:** 2026-03-23

**Authors:** Agnieszka Wojskowicz, Piotr Skalij, Dominika Hempel, Łukasz Zalewski, Monika Konopka-Filippow, Iwona Sidorkiewicz, Agnieszka Krzystyniak, Ewa Sierko

**Affiliations:** 1 Department of Radiotherapy I, Maria Skłodowska-Curie Białystok Oncology Center, 15-027 Bialystok, Poland; dhempel@onkologia.bialystok.pl (D.H.); lzalewski@onkologia.bialystok.pl (Ł.Z.); mkonopka@onkologia.bialystok.pl (M.K.-F.); esierko@onkologia.bialystok.pl (E.S.); 2 Department of Oncology, Medical University of Białystok, 15-027 Bialystok, Poland; pskalij@onkologia.bialystok.pl; 3 Department of Clinical Oncology, Maria Skłodowska-Curie Białystok Oncology Center, 15-027 Bialystok, Poland; 4 Clinical Research Centre, Medical University of Bialystok, 15-276 Bialystok, Poland; 5 Department of Medical Physics, Maria Skłodowska-Curie Białystok Oncology Center, 15-027 Bialystok, Poland

**Keywords:** immunotherapy, durvalumab, chemoradiotherapy, stage III, unresectable, NSCLC, elderly

## Abstract

**Background**: Non-small cell lung cancer (NSCLC) constitutes about 80–85% of lung cancers, and ~60–70% of NSCLC patients are diagnosed at an advanced stage of the disease. Concurrent chemoradiotherapy (cCRT) followed by consolidation durvalumab has become the standard of care for unresectable stage III NSCLC, following the phase III PACIFIC trial, which demonstrated significantly improved progression-free survival (PFS) and overall survival (OS) with durvalumab. **Methods**: We conducted a single-center retrospective study with unresectable stage III NSCLC patients who received cCRT between January 2021 and December 2025 at the Białystok Oncology Center (Poland). Patients with an ECOG performance status of 0–1 and no disease progression (PD) after cCRT were treated with durvalumab consolidation up to 12 months or until PD or unacceptable toxicity. The clinical outcomes and treatment tolerance were analyzed. **Results**: Out of 94 cCRT-treated patients (pts), 78 received durvalumab consolidation therapy. The median age of the pts was 66.5 years; 64.1% pts were >65 years old. Squamous carcinoma was the predominant histology (56.4%). The median time from cCRT completion to durvalumab initiation was 45 days (range: 15–85). A majority (57.7%) of patients completed the full 12 months of durvalumab. With a median follow-up of 40 months, the median PFS was ~1224 days (40.2 months). At 3 years, PFS was 52.8%. There were no significant differences in PFS by age (<65 vs. ≥65 years), HR:0.65, clinical stage (IIIA vs. IIIB/IIIC) HR:1.01, histology (squamous vs. non-squamous carcinoma), HR:0.76; sex HR:0.6, ECOG 0 vs. 1 HR:0.82; or initiation of durvalumab ≤42 vs. >42 days after cCRT, HR:0.62 (*p* > 0.05 for all). The sole factor significantly affecting PFS was smoking status: ever-smokers had a longer PFS than never-smokers (median ~46 months vs. ~21 months, HR:2.11, *p* = 0.04). Durvalumab consolidation was generally well tolerated. Grade 3–4 adverse events (mainly pneumonitis and esophagitis) leading to permanent durvalumab discontinuation occurred in 7 patients (9%), almost all over 65 years old. **Conclusions**: Real-world data from our single-center study confirm that consolidation durvalumab therapy after cCRT provides substantial clinical benefit in unresectable stage III NSCLC, even in older patients. The PFS and safety outcomes in our cohort, which had a higher proportion of elderly and locally advanced cases, were comparable with those reported in clinical trials (PACIFIC) and observational studies (PACIFIC-R), underscoring the effectiveness and tolerability of this approach in routine practice. We acknowledge the limitations of the retrospective design and sample size, but our findings support the use of cCRT followed by durvalumab in eligible stage III NSCLC patients and highlight the need for further research on optimizing outcomes (e.g., the impact of smoking and other biomarkers).

## Simple Summary

The PACIFIC study results have necessitated a new standard of care for patients with non-small cell lung cancer (NSCLC). We assessed the safety and real-world efficacy of durvalumab maintenance therapy after concurrent chemoradiotherapy (cCRT) in unresectable, locally advanced non-small cell lung cancer (NSCLC). All consecutive patients with unresectable stage III NSCLC were included in the study, regardless of PD-L1 expression. Median PFS for the entire study population was ~43.7 months (approximately 3.6 years). A total of 39 patients (50%) had disease progression or death at the end of follow-up. We achieved similar PFS results to those in the PACIFIC and PACIFIC-R trials, with acceptable toxicity (grade 3 or 4 adverse events occurred in 9% of patients, almost exclusively in those over 65 years of age). Progression was more common in the younger age group than in those over 65, while deaths were more common in older patients. The results of durvalumab maintenance therapy after cCRT in LA-NSCLC in daily practice confirm the effectiveness of the therapy in real-world settings, even in older patients.

## 1. Introduction

With the growing number of cancer patients, the number of elderly people undergoing cancer treatments increases, which is a result of the aging of populations in the European Union (EU) and the increase in average life expectancy. In the last 20 years, the proportion of people aged 65 and above in EU countries rose from 16.4% to 21.6% [1]. A similar demographic shift is occurring in Poland, where in 2024 people aged 60 and over constituted 26.6% of the total population [2]. The incidence of malignancies rises exponentially with age, and cancers are currently the second leading cause of death in Poland (after cardiovascular diseases), accounting for about one-quarter of all deaths [3]. Lung cancer remains the leading cause of cancer-related mortality worldwide for both women and men, with over 2 million new cases diagnosed annually [4,5]. The situation is similar in Poland, largely because the disease is often diagnosed too late. Each year in Poland, around 23,000 new lung cancer cases are registered, and almost the same number of patients die annually [6]. Lung cancer is a heterogeneous group of malignancies, consisting of non-small cell lung cancer (NSCLC) and small cell lung cancer (SCLC). The most common NSCLC subtypes are adenocarcinoma, squamous cell carcinoma, and large cell carcinoma [7].

NSCLC accounts for approximately 80–85% of all lung cancer cases [8], and 60–70% of NSCLC patients present locally advanced disease at diagnosis [9]. About one-third of NSCLC patients have stage III disease at the time of diagnosis [10]. Depending on primary tumor size/extent (T) and regional lymph node involvement (N), stage III is further classified into IIIA, IIIB, and IIIC. The treatment of choice for these patients may include surgery (if feasible) plus adjuvant immunotherapy or chemotherapy, or chemoradiotherapy (CRT), or radiotherapy (RT) alone [11,12,13]. It has been shown that RT alone is less effective than concurrent chemoradiotherapy (cCRT) or sequential chemoradiotherapy (sCRT) [14]. In both sCRT and cCRT, a total dose of 60–66 Gy in 30–33 fractions is typically delivered to the primary tumor and involved hilar/mediastinal lymph nodes, combined with 2–4 cycles of platinum-based chemotherapy [15,16,17]. In a 2010 meta-analysis by Aupérin et al. [10] (1205 NSCLC patients), concurrent CRT significantly improved overall survival (OS) and reduced the risk of locoregional progression compared to sequential therapy. However, cCRT is associated with a higher risk of acute toxicities, mainly esophagitis, pneumonitis, and hematologic complications [10]. Despite the improved outcomes with combined modality therapy over RT alone, the clinical results after CRT have remained unsatisfactory: the median progression-free survival (PFS) is only ~8 months, and the 5-year survival rate is barely 15% [10,18].

Therefore, research over recent decades has focused on methods to enhance treatment efficacy for stage III NSCLC [19,20,21]. A breakthrough came with the PACIFIC phase III trial, which combined cCRT followed by consolidation durvalumab. Durvalumab is a selective, high-affinity, human immunoglobulin G1 kappa (IgG1κ) monoclonal antibody that blocks programmed death ligand 1 (PD-L1) biding to programmed death 1 (PD-1) and CD80, allowing T cells to recognize and kill tumor cells [22,23]. The randomized PACIFIC trial showed that adding durvalumab after cCRT (at 10 mg/kg every 2 weeks for up to 1 year) significantly prolonged PFS and OS compared to placebo [24]. Patients were eligible to immunotherapy if they had unresectable stage III NSCLC with no disease progression after cCRT. A total of 713 patients with good performance status (ECOG 0–1) were enrolled. The median PFS was 16.8 months in the durvalumab group vs. 5.6 months in the placebo group. The 12-month PFS rates were 55.9% vs. 35.3%, and 18-month PFS rates were 44.2% vs. 27.0%, respectively. Treatment was well tolerated; grade 3/4 adverse events occurred in 32.6% (155/475) of patients receiving durvalumab vs. 28.2% (66/234) on placebo. The incidence of fatal adverse events was low and similar between durvalumab (4.4%) and placebo (6.4%) groups [25]. After 3 years of follow-up, 57% of patients in the durvalumab arm were still alive, compared to 43.5% in the placebo arm [26]. Notably, the 5-year OS rate was 42.9% in the durvalumab group, and 33.1% of patients were no signs of disease progression [27]. The PACIFIC trial changed the standard of care for stage III NSCLC patients.

On 16 February 2018, the U.S. Food and Drug Administration approved durvalumab for stage III NSCLC patients without progression after cCRT, and on 21 September 2018, the European Medicines Agency authorized its use in the EU. In Poland, full access to durvalumab therapy began on 1 January 2021 (National Drug Program annex B6).

The aim of this study was to retrospectively analyze the clinical effectiveness and tolerability of consolidation durvalumab in stage III NSCLC patients after cCRT at a single oncology center.

Since widespread access to consolidation therapy with durvalumab after cCRT became available in Poland, we analyzed all patients treated with cCRT in our center. Patients received concurrent radiotherapy with two courses of chemotherapy, preceded by one or two courses of induction chemotherapy (cisplatin or carboplatin with either vinorelbine or pemetrexed). All patients underwent thoracic radiotherapy to a dose of 60 Gy (±10%) in 2 Gy fractions. Patients who completed cCRT without progression and with ECOG 0–1 were eligible to receive the durvalumab consolidation therapy and were included in the analysis. The analysis considered the influence of factors on PFS, including age, clinical stage, histological type of cancer, gender, performance status, time from cCRT completion to durvalumab initiation and smoking status. This paper descibes retrospective analysis of real world data from everyday clinical practice whiche may help other clinical to qualification and treat the CS III NSCLC patients with durwalumab after cCRT.

## 2. Materials and Methods

### 2.1. Study Population

We reviewed medical records of 94 patients with unresectable stage III NSCLC who received cCRT between 1 January 2021 and 31 December 2025 at the Białystok Oncology Center in Poland. Most patients had been initially diagnosed at the 1st or 2nd Department of Pulmonary Diseases, Lung Cancer and Internal Medicine of Medical University in Białystok, Poland. Clinical staging was determined according to the 8th edition of TNM classification, with 43 patients classified as stage IIIA, 47 as IIIB, and 4 as IIIC [28].

All patients were in good general condition (ECOG performance status 0 or 1) at the time of treatment qualification. PD-L1 expression was not routinely assessed during durvalumab eligibility screening. The EGFR and ALK status was not evaluated. Baseline pulmonary function (FEV1 ≥ 1.0 L) were assessed in all patients and each patient underwent a cardiology consultation before qualification to combined therapy [29]. The most common comorbidities were chronic obstructive pulmonary disease (COPD), hypertension, heart failure, and type 2 diabetes. All patients received platinum-based chemotherapy (cisplatin or carboplatin) with either vinorelbine or pemetrexed. All patients underwent thoracic radiotherapy with the VMAT technique using six MV X-rays, to a dose of 60 Gy (±10%) in two Gy fractions. Two patients died during the cCRT.

Of the remaining 92 patients, 12 were not qualified for durvalumab consolidation therapy due to disease progression or deterioration in performance status after cCRT (ECOG 3–4). An additional two patients declined durvalumab treatment.

Thus, 78 patients who completed cCRT without progression and with ECOG 0–1 were eligible to receive the durvalumab consolidation therapy and were included to the analysis. The inclusion criteria are presented in (Table 1). The required criteria must be met cumulatively.

At durvalumab initiation, all patients had no contraindications to immunotherapy. Durvalumab was administered intravenously (20 mg/kg every 4 weeks, or 10 mg/kg every 2 weeks) for up to 12 months or until radiologically confirmed progression or presence of unacceptable toxicity. The median follow-up time from the end of cCRT was 40 months. Clinical and laboratory data were collected retrospectively from medical records. The progression-free survival (PFS) and the impact of various factors on disease progression were analyzed.

During durvalumab consolidation therapy, patients underwent monthly basic laboratory tests (blood counts, creatinine, liver enzymes) and, every 3 months, imaging (CT of the chest, abdomen, and pelvis), electrocardiogram, and thyroid function tests (TSH, fT3, fT4).

After completing durvalumab, patients remained under surveillance with CT scans of the chest, abdomen and pelvis every 3 months for the first 2 years, then every 6 months thereafter.

Treatment-related adverse events (AEs) during durvalumab therapy were graded using CTCAE v4.0 [30].

**Scheme 1:**
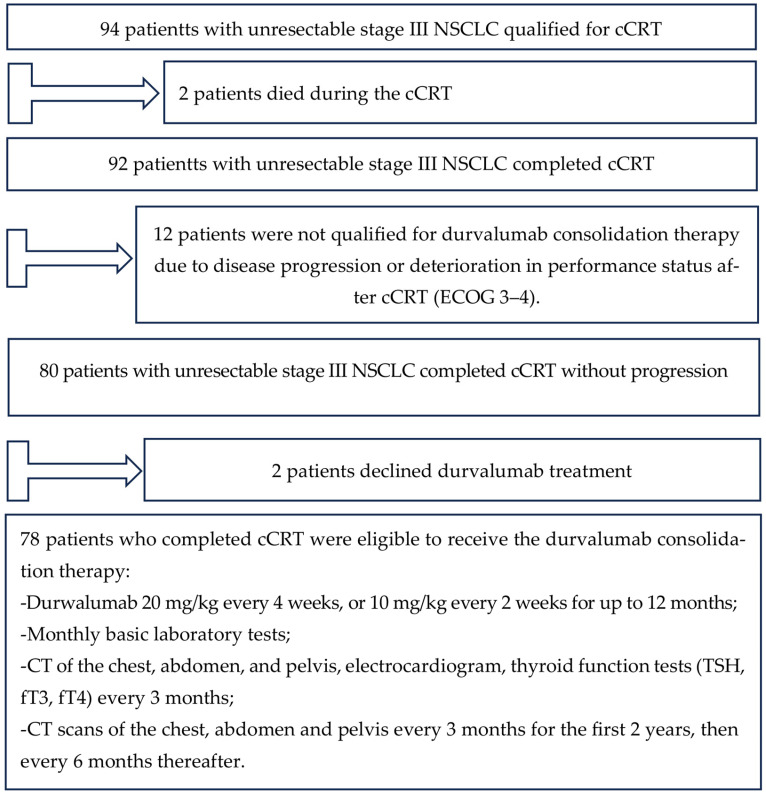
Bioethics Committee approval was obtained from the Medical University of Białystok (permit no. APK.002.376.2023).

The general scheme of the study design is presented in Scheme 1.

### 2.2. Statistical Analysis

Statistical analysis was performed using a significance level of α = 0.05. This means that we accepted a 5% probability of incorrectly rejecting the true null hypothesis, which is equivalent to a risk of a Type I error.

For categorical variables, results are presented as counts (n) and the percentage of each category.

To analyze the time to event (Progression-Free Survival, PFS), which was defined as the time from the end of cCRT to the date of radiologically confirmed disease progression or death, we used the Kaplan–Meier method, a nonparametric technique that allows for the estimation of survival functions while accounting for censored data. Kaplan–Meier survival curves were used to graphically display the data, enabling intuitive comparison of survival functions across study groups.

Comparison of survival times between study groups was performed using the log-rank test.

To assess the impact of various progression markers on the efficacy of durvalumab treatment, we used a Cox regression model (univariate and multivariate). The analysis focused on the assessment of the hazard ratio (HR) and its corresponding 95% confidence intervals (CI). Statistical analyses were performed using GraphPad Prism v9.5.1.

## 3. Results

The final analysis included 78 patients who received durvalumab consolidation therapy. Women comprised 33.3% (n = 26) and men 66.7% (n = 52) of all patients. Patient age ranged from 38 to 77 years (median 66.5 years). Only 28 patients (35.9%) were ≤65 years old, whereas 50 patients (64.1%) were >65. Nineteen patients (24.3%) were over 70 years of age. ECOG performance status was 0 in 44 patients (56.4%) and 1 in 34 patients (43.6%). Regarding stage distribution, 35 patients (44.9%) had stage IIIA, 40 patients (51.3%) stage IIIB, and 3 patients (3.8%) stage IIIC of the disease. The dominant histopathological subtype was squamous cell carcinoma (44 patients, 56.4%), followed by adenocarcinoma (27 patients, 34.6%). A history of tobacco use was noted in the majority of patients: 30 were former smokers and 33 were active smokers at the time of treatment. The total radiation dose was >60 Gy in 88.5% of patients; only 9 patients (11.5%) received a lower dose (54–60 Gy). The most common chemotherapy regimen was vinorelbine plus platinum (89.7% of patients). The median interval from cCRT completion to the start of durvalumab was 45 days (range: 15–85 days). Most patients (n = 36, 46.2%) received the first durvalumab dose between day 43 and day 60 after cCRT, while 34 patients (43.6%) started durvalumab within 42 days of cCRT completion. Of the 78 patients, 45 (57.7%) completed the planned 12 months of durvalumab therapy. The median duration of durvalumab treatment was ~10.1 months (307.5 days; range: 1–384 days). At the time of data cutoff, 2 patients (2.6%) were still on durvalumab treatment (Table 2).

The most common AEs observed were pneumonitis (or interstitial lung disease), with a similar frequency in younger and older patients, and esophagitis, occurring more frequently in the intermediate age group: 65–69 years. Seven patients (9%) experienced grade 3 or 4 AEs, leading to early termination of durvalumab (Table 3). Importantly, 6 of these 7 patients were over 65 years old (including 2 patients over 70). During the observation period, we did not observe any immunological complications such as hepatitis, enteritis, hypothyroidism, diarrhea, adrenal insufficiency, or skin lesions.

In total, durvalumab was discontinued in 31 patients (39.7%) due to disease progression, AEs, or deterioration in performance status (Table 4).

By the end of follow-up, disease progression was documented in 28 patients (35.8%) who had received at least one dose of durvalumab (in 38.5% of treated women and 34.6% of treated men). Progressive disease was observed more frequently during durvalumab treatment than after finishing the immunotherapy (22/28 patients).

Among patients <65 years old, 42.9% experienced progression, whereas in those ≥65 years old, 32% had PD.

At the time of analysis, 31 patients (39.7%) had died—9 out of 26 women (34.6% of all women) and 22 out of 52 men (42.3% of all men) who received durvalumab consolidation. Death was reported more frequently in the group of patients older than 65 compared to younger patients (23.1% vs. 11.5%, respectively). A total of 11 patients (39.3%) died in the age group <65 years; 13 patients (41.9%) died in the 65–69 years group, and 7 patients (36.8%) died in the group >70.

The primary endpoint of the analysis was progression-free survival (PFS). The median PFS for the entire cohort was approximately 1224 days (40.2 months, or 3.35 years). By the end of follow-up (31 December 2025), 39 patients (50% of the cohort) experienced disease progression or died.

**Figure 1:**
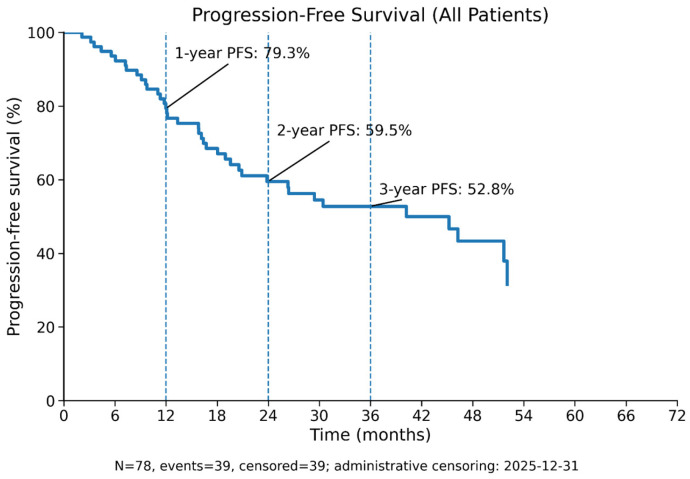
Progression-free survival (PFS) for all patients.

**Figure 2:**
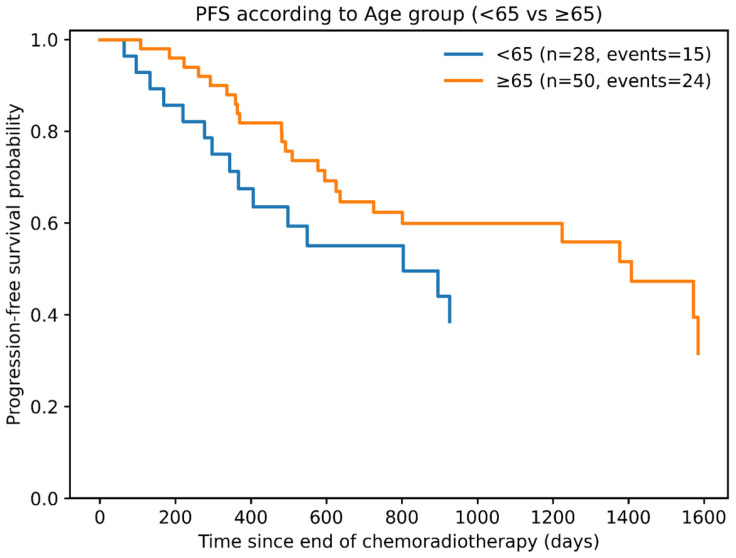
Progression-free survival (PFS) according to age group (<65 vs. ≥65 years).

**Figure 3:**
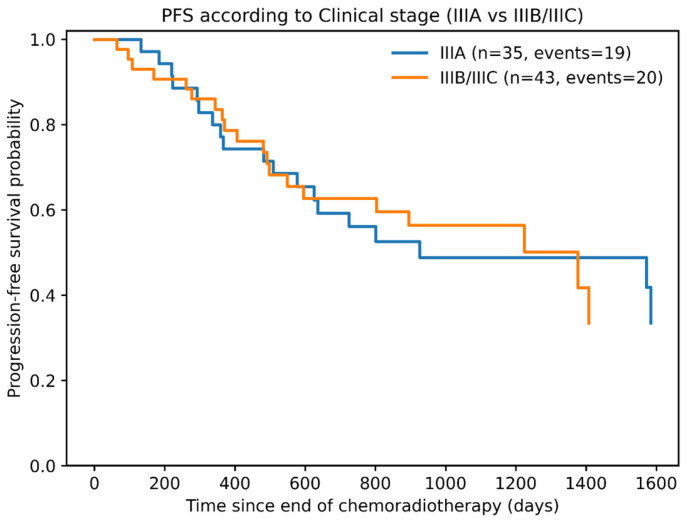
Progression-free survival (PFS) according to clinical stage (IIIA vs. IIIB/C).

**Figure 4:**
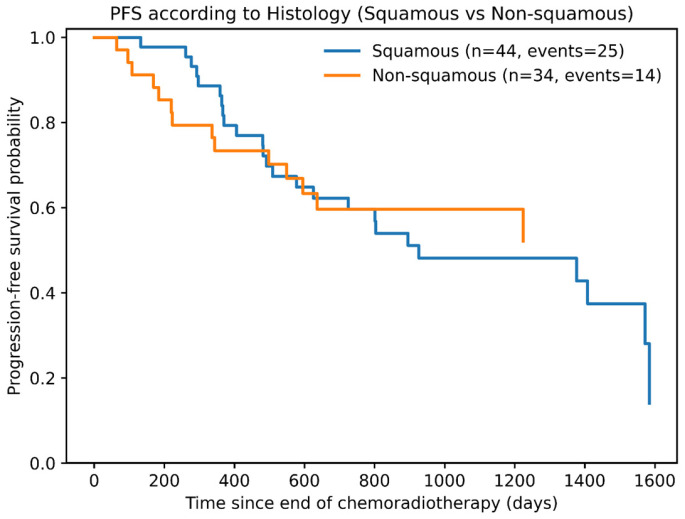
Progression-free survival (PFS) according to histology (squamous vs. non-squamous).

**Figure 5:**
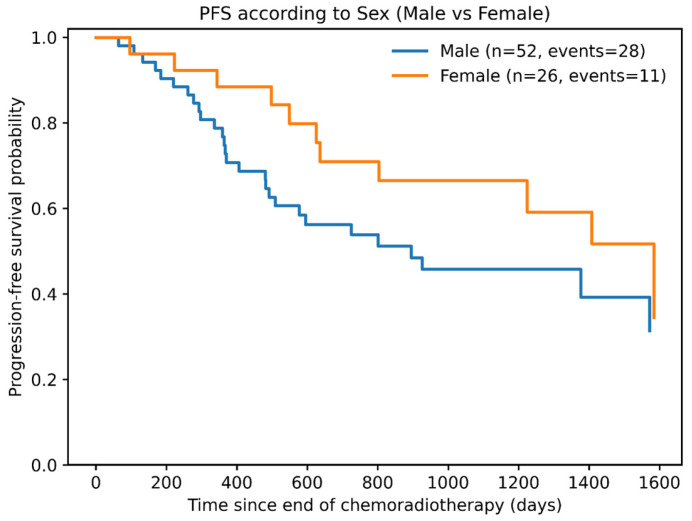
Progression-free survival (PFS) according to sex (male vs. female).

**Figure 6:**
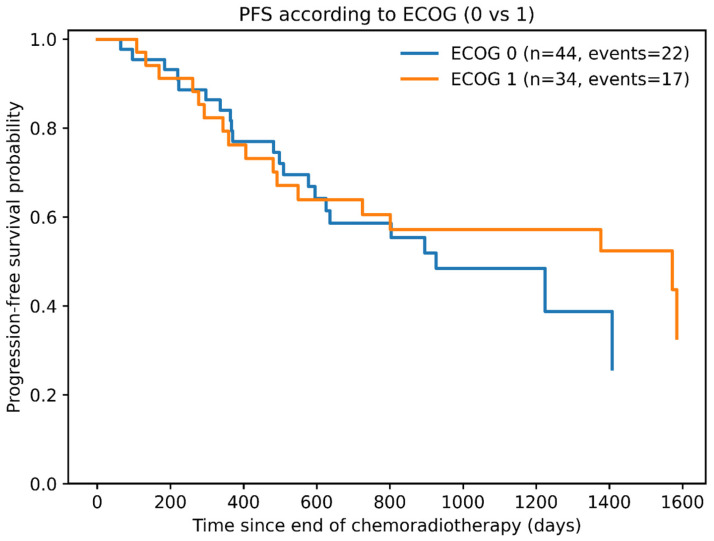
Progression-free survival (PFS) according to ECOG (0 vs. 1).

**Figure 7:**
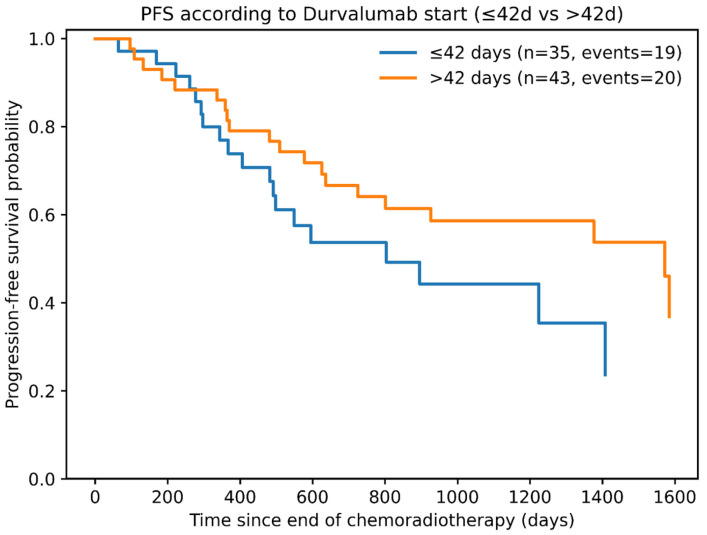
Progression-free survival (PFS) according to durvalumab start (≤42 d vs. >42 d).

Kaplan–Meier analysis of PFS is shown in Figure 1 (overall population). No significant impact on PFS was observed for age (<65 vs. ≥65 years) (Figure 2), clinical stage (IIIA vs. IIIB + IIIC) (Figure 3), histological subtype (squamous vs. adenocarcinoma) (Figure 4), sex (Figure 5), performance status (ECOG 0 vs. 1) (Figure 6), or interval from cCRT to durvalumab initiation (≤42 days vs. >42 days) (Figure 7). In the PD-L1 testing subset, we found no imbalance in PD-L1 expression between smoking groups: the PD-L1(+) rate was 37.0% (10/27) in ever-smokers and 33.3% (2/6) in non-smokers. Fisher’s exact test was nonsignificant (*p* = 1.00), and the odds ratio for PD-L1(+) (ever vs. never) was 1.18 (95% CI 0.14–15.16). This does not support the hypothesis that the observed effect of smoking on PFS is due to a simple “shift” of the PD-L1 distribution toward higher expression in smokers. In Cox models in this subset, including PD-L1 did not significantly alter the effect of smoking on PFS: the HR for smoking was 0.54 in the univariate model and 0.49 after adjusting for PD-L1. At the same time, PD-L1(+) showed a trend toward longer PFS (HR = 0.24; *p* = 0.068), but the analysis was limited by the small number of events and wide confidence intervals.

**Figure 8:**
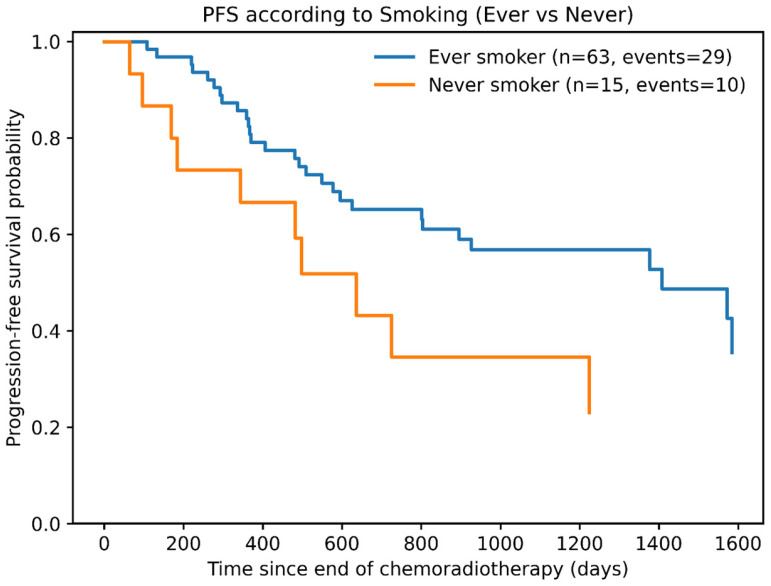
Progression-free survival (PFS) according to smoking (ever vs. never).

A multivariate Cox model confirmed that none of these variables independently influenced the risk of progression. The only factor that significantly affected PFS in both univariate and multivariate analysis was smoking status: patients with a history of smoking had significantly longer PFS than never-smokers (Figure 8; Table 5).

## 4. Discussion

This analysis is a retrospective, single-center observational study of patients with unresectable stage III NSCLC who received consolidation durvalumab therapy after cCRT in a real-world setting. In routine practice, only patients without severe comorbidities (e.g., uncontrolled respiratory or cardiac failure, recent myocardial infarction), with good performance status (ECOG < 2), without active infection, >10% unintended weight loss in the last 3 months and significant pleural effusion are considered for this aggressive combined approach [10,11]. All patients in our cohort were qualified for cCRT and durvalumab by a multidisciplinary tumor board. De Castro Jr. et al. [31] showed in a meta-analysis that multidisciplinary team (MDT) care is associated with longer OS of the treated patients. Unlike the strictly selected population of a randomized trial, this study reflects outcomes in patients treated under real-world conditions.

The demographic characteristics of the patients were generally similar to those in the pivotal PACIFIC trial [24] and the real-world PACIFIC-R study [32]. The majority of patients were male and current or former smokers. This profile of patients also resembles that of the retrospective SPOTLIGHT study of patients receiving durvalumab consolidation after cCRT, where most patients were male, 95.2% had a smoking history, and the median age was 67.5 years (60.8–74.0) [33]. In our study, a larger proportion of patients (64.1%) were over 65 years old, whereas in the PACIFIC trial, more than half (54.8%) were under 65. Notably, the PACIFIC trial did include even very elderly patients (age range 23–90) [24]. The PACIFIC-R study [32] also enrolled a substantial number of older patients: 21.2% of participants were 70–75 years old, and 10.4% were 75 or older. However, in PACIFIC-R, a higher percentage of patients aged 70 years or older received sequential CRT (sCRT) rather than concurrent CRT (cCRT) (40.8% vs. 29.0%) [32]. In this study, only 24.3% of patients were above 70 years old. We did not offer cCRT to patients above 77 years of age at our center. This cautious approach to very elderly patients aligns with expert recommendations that stage III NSCLC patients over 70 years old, even with adequate cardiorespiratory function and without serious comorbidities, should be qualified to receive sCRT rather than cCRT [34]. Although age over 65 has been identified as an independent adverse prognostic factor in some studies [35,36], in this analysis, patient age did not significantly impact PFS. These results suggest that cCRT followed by durvalumab can be considered even for elderly patients. A post hoc analysis of PACIFIC using a 70-year age cutoff demonstrated that durvalumab after cCRT improved PFS and OS in patients ≥70 years, despite a higher incidence of grade 3/4 AEs than in placebo recipients in that age group [37].

The time from completing cCRT to starting durvalumab exceeded 42 days in 56.4% of patients in this study. However, the delay was not associated with worse PFS. A Canadian meta-analysis similarly showed that initiating durvalumab beyond 42 days after CRT did not adversely affect OS [38]. Some studies have suggested that patients achieve maximal benefit if consolidation therapy is given as soon as possible after radiotherapy [24,32]. Radiotherapy induces immunomodulatory changes, like increased tumor PD-L1 expression, which potentially enhance responsiveness to durvalumab [39,40,41]. However, in real-world practice, it is often challenging to start durvalumab within the recommended timeframe, as confirmed by the PACIFIC-R registry [32]. In PACIFIC-R, the median time to durvalumab initiation was 56 days post-CRT, and only 30.1% of patients began durvalumab within 42 days [32]. Similarly, in a multi-center Italian study, 78% of patients received durvalumab more than 42 days after CRT, with a median interval of 52 days (range 9–245) [42]. The authors of the Italian study [43] emphasize that the decision to commence durvalumab should be based on clinical assessment of the patient’s performance status and resolution of CRT-related toxicities.

In this analysis, the majority of patients receiving durvalumab consolidation therapy had squamous cell carcinoma. Various studies have shown that this histology is associated with worse prognosis compared to non-squamous NSCLC [32,38,42]. In both the PACIFIC trial and the PACIFIC-R study [24,32], non-squamous carcinoma cases were more common. Nevertheless, our analysis did not find a significant difference in PFS between squamous and non-squamous carcinoma patients.

In the present study, patients with a performance status worse than ECOG 1 for cCRT were not qualified for the treatment, whereas PACIFIC-R did include some patients with ECOG 2 [32]. In our cohort, 43.6% of patients were ECOG 1, but this did not significantly influence PFS. Other studies indicate that performance status correlates with treatment response, tolerance, quality of life, and OS [44,45,46].

It is noteworthy that despite a substantial proportion of high-risk patients with stage IIIB/IIIC disease (55.1% of the cohort)—which is associated with a greater risk of progression [40]—the outcomes were comparable in this study with the PACIFIC trial. In the PACIFIC trial, the stage IIIA patients were the largest group (52.9%).

Smoking status emerged as a significant prognostic factor for PFS. Patients with a history of smoking had markedly longer PFS than never-smokers (median ~46 vs. ~21 months, *p* = 0.04). It has been shown that tobacco smoke and its polycyclic aromatic hydrocarbons cause DNA damage and carcinogenesis, increase the mutational burden, and induce PD-L1 expression on bronchial epithelial cells [47,48]. This could result in an improved response to immune checkpoint inhibitors (ICIs), including durvalumab, in smokers [49,50].

In our study, over half (52.6%) of the patients received one or two cycles of induction chemotherapy (ICT) before cCRT. Likewise, in PACIFIC-R, 48.4% of patients had ICT, and in PACIFIC, 27% received ICT. While induction chemotherapy is not routinely recommended due to toxicity [51], in practice, this approach can help avoid delays in starting definitive therapy by giving the time required for RT planning [43]. Spigel et al. [52] found that in the PACIFIC trial, patients who received ICT experienced fewer treatment-related serious adverse events (SAEs) and less pneumonitis, regardless of treatment arm.

All patients in our series received platinum-doublet chemotherapy (cisplatin or carboplatin if cisplatin was contraindicated) combined with vinorelbine or pemetrexed, as these regimens are preferred for CRT. Cisplatin has been shown to prolong survival more effectively than carboplatin in the curative-intent treatment of NSCLC [53,54].

It is common knowledge that a higher prevalence of comorbidities comes with advanced age. Ruysscher et al. noted that more than half of stage III NSCLC patients would, in theory, not qualify for cCRT due to advanced age or comorbid conditions [55]. In a study by Asmis et al. involving 1255 NSCLC patients treated with chemotherapy, it was comorbidity burden—not age over 65—that predicted worse outcomes [36]. In our study, as in PACIFIC and PACIFIC-R, patients commonly had comorbidities such as COPD, cardiovascular disease, and diabetes [24,32], likely reflecting the impact of tobacco use.

In Poland, durvalumab consolidation is administered for a maximum of 12 months, or less if patients develop unacceptable toxicity or disease progression. In our cohort, 45 patients (57.7%) received the full 12 months of durvalumab. The median duration of durvalumab therapy in this study was slightly longer than in PACIFIC (43.9 weeks vs. 40.1 weeks).

After 3 years of follow-up, our study showed a better PFS rate (52.8%) compared to PACIFIC-R (42.2%) and PACIFIC (39.7%) studies [56]. This may have been due to greater caution in selecting patients for cCRT. All qualified patients remained in good or very good clinical condition. Furthermore, all patients were carefully prepared for internal disease: they underwent cardiology and pulmonology consultations, including possible treatment modifications, and a dietary consultation (a high-protein diet and ONS were recommended), which could have influenced the final treatment outcomes [57,58]. Patients were instructed to stop smoking, which has been shown to improve overall treatment outcomes [59]. More than half of the patients (41 patients; 52.56%) received a total of 3 or 4 chemotherapy cycles due to the use of induction chemotherapy (52.6%), which could have translated into a greater reduction in lung tumor volume and, therefore, better PFS. Greater tumor volume reduction after induction chemotherapy enabled the administration of a higher total radiotherapy dose (>60 Gy; 69 patients; 88.5%). However, D. Spigel et al. in a subgroup analysis of the PACIFIC study did not demonstrate that the use of induction chemotherapy or a higher dose of RT translated into better PFS or ORR [52]. To date, the issue of the ICT and high dose of RT (66Gy versus 60Gy versus 54Gy) is unclear. Disease progression was noted more frequently in women (38.5%) than in men (34.6%), and in the younger subgroup (42.9% for <65) than in the older subgroup (32% for ≥65) of patients. However, these differences were not statistically significant. Conversely, mortality was observed more often in patients over 65 than in younger patients (23.1% vs. 11.5%).

PD-L1 expression status was not assessed in as many as 57.7% of our patients, likely because PD-L1 status is not a mandatory criterion for durvalumab reimbursement in Poland’s drug program B6. Some studies have indicated that patients with PD-L1 expression ≥ 1% achieve greater benefit from durvalumab consolidation than those with PD-L1 < 1% [32,60]. However, other real-world studies, such as the Spanish S-REAL [61] and a German study [62], found no significant impact of PD-L1 status on OS or PFS, questioning the exclusion of PD-L1–negative patients from durvalumab consolidation. This discrepancy could be due to tumor heterogeneity (different PD-L1 expression across tumor samples) and the possibility that radiotherapy may enhance tumor response to immunotherapy [39,40,41,63].

In terms of safety, our results were satisfactory. Durvalumab was permanently discontinued due to severe AEs in 9% of our patients, whereas treatment was stopped because of toxicity in 15.4% of PACIFIC patients and in 16.5% of PACIFIC-R patients [24,32]. In our study, nearly all patients who discontinued durvalumab therapy due to AEs were over 65 years old. Similarly, a Korean study [64] reported a higher incidence of durvalumab-related adverse events in elderly patients (above 70 years).

## 5. Limitations of the Study

We are aware of the limitations of our analysis because we collected information retrospectively after the events occurred, an analysis that was not planned a priori. Furthermore, the retrospective nature of the study leads to some missing data; for example, the lack of PD-L1 assessment in a group complicates the interpretation of treatment results. Due to the nature of the study, we had no control over confounding variables that could have influenced the final results and were not recorded in the documentation. A significant problem is the representativeness of the study group. The number of patients in our analysis was relatively small (78), reflecting the single-center nature of the study. A small group of patients may lead to a low statistical value. On the other hand, the study is from the largest cancer center in northeastern Poland, where therapeutic decisions are made by multidisciplinary teams. We would like our results to encourage further prospective, multicenter studies.

## 6. Conclusions

In summary, our real-world findings complement clinical trial results and confirm the benefit of durvalumab consolidation therapy in patients with unresectable stage III NSCLC who do not progress after cCRT, including the elderly. Despite our cohort having higher-risk features at baseline (older age, more advanced stage, higher proportion of squamous carcinoma), we achieved outcomes comparable to those of the PACIFIC and PACIFIC-R studies, and the safety profile of durvalumab was similar or even more favorable. We are aware that our results should be interpreted in light of the study’s limitations, particularly the small sample size and the single-center, retrospective design. Nonetheless, our experience reinforces the use of the cCRT plus durvalumab paradigm in routine practice and underscores the importance of continuing efforts to optimize therapy for stage III NSCLC.

## Figures and Tables

**Table 1:** Inclusion criteria to treatment with durvalumab.

Inclusion Criteria
A histological or cytological diagnosis of non-small cell lung cancer (all types)
Unresectable stage III disease—patients who have not progressed following platinum-based radical concurrent chemoradiotherapy (CT scans performed within 6 weeks of finishing radiotherapy)
A performance status of 0–1 according to the Zubrod-WHO or ECOG classification
Age over 18 years
No other uncontrolled comorbidities
No active autoimmune diseases excluding diabetes, hypothyroidism, psoriasis, or vitiligo
Adequate blood, kidney, and liver function
Exclusion of contraindications to durvalumab listed in the SPC
No malignances uncontrolled by treatment

SPC—Summary of Product Characteristics.

**Table 2:** Patient demographics, disease characteristics, treatment characteristics.

Study Population (n = 78)	
Median age (years)	66.5
Range age (years)	38–77
Age groupings, n (%)	
<65	28 (35.9)
65–74	46 (59)
75+	4 (5.1)
Sex, n (%)	
Female	26 (33.3)
Male	52 (66.7)
Stage, n (%)	
IIIA	35 (44.9)
IIIB	40 (51.3)
IIIC	3 (3.8)
Histology, n (%)	
Squamous cell carcinoma	44 (56.4)
Adenocarcinoma	27 (34.6)
Other	7 (9)
Smoking history, n (%)	
Former	30 (38.4)
Current	33 (42.3)
Never	15 (19.3)
ECOG performance status score, n (%)	
0	44 (56.4)
1	34 (43.6)
Chemotherapy regimens	
Induction (1 cycle)	15 (19.3)
Induction (2 cycles)	26 (33.3)
Concurrent	37 (47.4)
Chemotherapy regimens	
Vinorelbine + Cisplatin/Carboplatin	70 (89.7)
Pemetrexed + Cisplatin/Carboplatin	6 (7.7)
Etoposide + Cisplatin/Carboplatin	2 (2.6)
PD-L1 expression level	
positive	12 (15.3)
negative	21 (26.9)
unknown	45 (57.7)
Radiation total dose n (%)	
54–60 Gy	9 (11.5)
>60 Gy	69 (88.5)
Lung mean dose (Gy), median (range)	15.6 (7.1–27.1)
V20 lung sum (%) mean (range)	27.2 (10.9–53.2)
V5 lung sum (%) mean (range)	58.14 (30.0–97.6)
Heart mean dose (Gy), median (range)	4.9 (0.5–23.8)
Esophageal mean dose (Gy), median (range)	17.4 (4.3–46.0)
Time to durvalumab initiation from end of radiation therapy (days), median (range)	45 (15–85)
Time to durvalumab initiation from end of radiation therapy, n (%)	
0–13 days	0 (0)
14–27 days	8 (10.3)
28–42 days	26 (33.3)
43–60 days	36 (46.1)
60+ days	8 (10.3)
Duration of treatment with durvalumab (days) median	307.5
Patients with ongoing durvalumab treatment at the end of the study period, n (%)	2 (2.6)
Patients whose provider documented that durvalumab treatment was complete, n (%)	45 (57.7)

ECOG, Eastern Cooperation Oncology Group; PD-L1, programmed death-ligand 1; Gy, gray.

**Table 3:** Adverse events of any grade in patients with unresectable LA-NSCLC.

Event	Any Grade	Grade 3 or 4
Number of patients with an event n (%)		
Any event	70 (89.7)	15 (19.2)
Cough	22 (28.2)	4 (5.1)
Dyspnea	12 (15.3)	4 (5.1)
Pneumonitis or radiation penumonitis	16 (20.5)	4 (5.1)
Esophagitis	15 (19.2)	0
Bronchoesophageal fistula	1(1.3)	1(1.3)
Myocardial infarction	1(1.3)	1(1.3)
Myositis	1(1.3)	1(1.3)
Hyperthyroidism	2 (2.6)	0

**Table 4:** Reasons for durvalumab treatment discontinuation in the group of 78 patients with unresectable LA-NSCLC.

Patients with Early Durvalumab Treatment Discontinuation, n (%)	31 (39.7)
Reasons for early durvalumab treatment discontinuation, n (%)	
Local progression	11 (14.1)
Distant progression (mts OUN, adrenal gland, muscles, axillary node)	11 (14.1)
Deterioration of overall condition according to ECOG	2 (2.6)
Pneumonia	4 (5.1)
Bronchoesophageal fistula	1 (1.3)
Myositis	1 (1.3)
Myocardial infarction	1 (1.3)

**Table 5:** Baseline preexisting predictors of PFS.

Patients with a Possible Predictor	Univariable			Multivariable		
	HR	95% CI	*p*-Value	HR	95% CI	*p*-Value
Age ≥ 65 vs. <65	0.65	0.34–1.24	0.194	0.61	0.30–1.24	0.176
Stage IIIB/IIIC vs. IIIA	1.01	0.53–1.92	0.971	0.91	0.44–1.90	0.803
Histopathology (squamous vs. non-squamous)	0.76	0.40–1.47	0.418	0.55	0.25–1.20	0.134
Sex (female vs. male)	0.60	0.30–1.21	0.154	0.56	0.26–1.20	0.138
ECOG 1 vs. 0	0.82	0.43–1.58	0.560	0.94	0.44–1.98	0.864
>42 days vs. ≤42 days	0.62	0.33–1.17	0.139	0.56	0.26–1.20	0.138
Smoking (never vs. ever)	2.11	1.02–4.38	0.044	2.90	1.25–6.76	0.013

## Data Availability

Data may be available upon reasonable request and with permission of the Medical University of Białystok in Poland.
